# Projected Heat-Related Mortality in the U.S. Urban Northeast

**DOI:** 10.3390/ijerph10126734

**Published:** 2013-12-03

**Authors:** Elisaveta P. Petkova, Radley M. Horton, Daniel A. Bader, Patrick L. Kinney

**Affiliations:** 1 Department of Environmental Health Sciences, Mailman School of Public Health, Columbia University, 722 West 168th St., New York, NY 10032, USA; E-Mail: epp2109@columbia.edu; 2 Columbia University Center for Climate Systems Research, 2880 Broadway, New York, NY 10025, USA; E-Mails: rh142@columbia.edu (R.M.H.); dab2145@columbia.edu (D.A.B.)

**Keywords:** heat-related mortality, climate change, New York, Boston, Philadelphia

## Abstract

Increased heat-related mortality is projected to be among the major impacts of climate change on human health, and the United States urban Northeast region is likely to be particularly vulnerable. In support of regional adaptation planning, quantitative information is needed on potential future health responses at the urban and regional scales. Here, we present future projections of heat-related mortality in Boston, New York and Philadelphia utilizing downscaled next-generation climate models and Representative Concentration Pathways (RCPs) developed in support of the Intergovernmental Panel on Climate Change (IPCC)’s Fifth Assessment Report (AR5). Our analyses reveal that heat-related mortality rates per 100,000 of population during the baseline period between 1985 and 2006 were highest in Philadelphia followed by New York City and Boston. However, projected heat-related mortality rates in the 2020s, 2050s and 2080s were highest in New York City followed by Philadelphia and Boston. This study may be of value in developing strategies for reducing the future impacts of heat and building climate change resilience in the urban Northeast region.

## 1. Introduction

Heat-related mortality is among the largest and most quantifiable of the expected impacts of climate change on human health [1]. Urban areas are particularly susceptible to the effects of heat due to the heat island effect, as well as large populations of vulnerable individuals [2]. The Northeastern U.S. urban corridor of New York City, Philadelphia and Boston is the largest population agglomeration in the country and among the largest in the World. Since the beginning of the 20th century, New York, Boston and Philadelphia have been experiencing a warming of 0.18, 0.15, and 0.13 degrees Celsius (°C) per decade, respectively. Prior studies have projected that average temperatures will increase by 3 to 5 Celsius (°C), by the last decades of the current century [3,4], together with an approximate doubling or tripling of the number of hot days each summer [5,6].

The Consortium for Climate Risk in the Urban Northeast (CCRUN) was created in 2010 under NOAA’s Regional Integrated Sciences and Assessments (RISA) Program with the mission to assist stakeholders from various sectors including health, in assessing and managing climate change impacts [7]. The impacts of climate change, and heat in particular, on health in the urban Northeast have become an issue of growing public concern in recent years [8,9]. With its current focus on Boston, New York and Philadelphia, one of CCRUN’s primary health-related objectives is to derive a comparative assessment of projected heat-related mortality across the three cities, in order to support decision maker efforts to reduce heat-related vulnerability.

Various studies to date have provided assessments of the potential future impacts of heat, projecting substantial increases in heat-related mortality due to climate change [10,11,12,13,14,15,16,17,18,19,20,21]. While some previous studies have investigated future heat-related mortality in Boston, Philadelphia and New York City [12,13,17,18], the utilization of different metrics and methodologies makes comparing assessments across cities challenging. In addition, the impacts of climate change on heat-related mortality under the next-generation Intergovernmental Panel on Climate Change (IPCC)’s Fifth Assessment Report (AR5) models [22] and Representative Concentration Pathways (RCPs) [23] are yet to be investigated in any of the cities of interest.

In this paper, we present the first estimates of heat-related mortality in Boston, New York City and Philadelphia based on downscaling of the new coupled global climate models and two of the Representative Concentration Pathways (RCPs), RCP4.5 and RCP8.5. We start by characterizing the heat-mortality relationships in each city based on 22 years of historical daily temperature and mortality data. Next, we present mortality projections based on the downscaled temperature projections. Heat-related mortality rates are used as the outcome measure. Finally, we calculate future heat-related deaths and mortality rates based on the ensemble of temperature projections.

## 2. Materials and Methods

We started by characterizing the summer heat-mortality relationships between observed daily mortality and mean temperature data in each city. The summer season was defined as the months of May, June, July, August and September. Daily all-cause mortality data for Boston, New York and Philadelphia from 1985 to 2006 were obtained in collaboration with Dr. Joel Schwartz and colleagues at Harvard University School of Public from the U.S. National Center for Health Statistics [24]. The following counties were included in the city-specific mortality data: Suffolk County, MA for Boston, New York County, NY, Kings County, NY, Queens County, NY, Bronx, NY and Richmond County, NY for New York City and Pennsylvania County, PA for Philadelphia. Daily mean temperature data for the same period for each city were obtained from the U.S. National Climatic Data Center [25].

The distributed lag non-linear module in R [26] was used to model the summer heat-mortality relationships. Models for each city were developed using natural cubic splines with four degrees of freedom for the temperature and the lag. We also fitted models with splines ranging from three to five degrees of freedom for the temperature and from three to five degrees of freedom for the lag and found that findings were robust to modeling parameters. We tested models with lags between one and seven days and found that lag duration of four days was sufficient to capture fully the heat effect in each location. Based on previous studies published in the literature [27,28], we used two splines to control for seasonality: a natural spline with two degrees of freedom per year to control for long term seasonal cycles, and a natural spline with four degrees of freedom for day in year to control for within summer seasonal variation. Data on ozone and particulate matter with aerodynamic diameter of 10 µm or less (PM10) were not included in the model since they were found to not substantially impact results in a previous study [18]. All models were developed using mean daily temperature and 20 °C as a reference temperature for calculating relative risk (RR) above 25 °C. Consistent reference temperatures and temperature thresholds as opposed to city-specify percentiles were used in estimating temperature effects. This approach allows quantifying and comparing the impact of an identical temperature exposure on mortality across the three cities.

Our spatial and temporal downscaling approach begins with monthly bias-corrected and spatially disaggregated (BCSD) climate projections at 1/8° resolution derived from the WCRP CMIP5 multi-model data set. BCSD projections were obtained online [29] for 33 global-scale general circulation models (GCMs) used in the Intergovernmental Panel on Climate Change (IPCC)’s Fifth Assessment Report (AR5), and two representative concentration pathways (RCPs) [23]. Detailed information about the 33 climate models is provided in Table S1. The new RCPs were developed for the climate modeling community as a basis for long-term and near-term climate modeling experiments in support of the IPCC AR5. RCPs, which replace the emissions scenarios [30] used in prior IPCC assessments, make various underlying assumptions about radiative forcing through time, which is dependent upon future global greenhouse gas and aerosol concentrations, as well as land use changes.

For this analysis, we selected the two RCPs most used by the climate modeling community, RCP4.5 and RCP8.5, which represent relatively low and high greenhouse gas projections/radiative forcing, respectively. RCP4.5 is a scenario where greenhouse gas concentrations are stabilized after 2100, due to emissions reduction prior to 2100. RCP8.5 is a scenario with increasing emissions over the century. Increasing emissions are associated with a highly energy intensive future, that features high population growth and slow development of green technologies such as renewable energy sources and energy efficiency [31].

The monthly output from the land-based 1/8° grid box corresponding to Boston (Airport), New York (Central Park), and Philadelphia (Airport) was then used to create change factors for each calendar month based on the difference between a 30-year future average (or ‘timeslice’) for that calendar month and the same GCM’s 30-year baseline average for that same calendar month [32]. We next applied the calendar-month change factors to the respective observed daily weather data for each of the three cities to create a future projection with the same statistical characteristics and sequence as the observations. Our downscaled output is a set of 66 weather station-specific synthetic future temperature projections for daily mean temperature in each city from 2010 to 2099 based on three 30-year time slices, defined as the 2020s (2010 to 2039), 2050s (2040 to 2069) the 2080s (2070 to 2099), and for a baseline period of 1971 to 2000.

The approach described here does not explore how intra-annual and inter-annual temperature variability may change. By not considering sub-monthly changes in variability, we were able to use fine-spatial-resolution projections (as the 1/8° BCSD product is monthly, not daily). By applying the delta method separately for each calendar month, we do capture one component of possible changes in intra-annual variance, changes in the annual temperature cycle. Previous studies have found changes in the annual cycle to be important [33].

The derived temperature-specific relative risk estimates for Boston, New York City and Philadelphia were applied to the daily downscaled temperature projections until 2100 for each city. Temperature curves were linearly extrapolated for temperatures up to 42 °C, the highest projected temperature, using the last four points of each curve. City-specific estimates of annual summer heat-related mortality were computed as described below.

Our approach to calculating heat-related mortality was similar to that presented in a previous study [34]. First, using the temperature-specific relative risks derived from the models for each city, we calculated historical heat-related attributable risk and projected heat-related attributable risk for temperatures 25 °C and above. Daily observed temperatures were used in calculating the historical and daily downscaled temperature projections were used for calculating future heat-related attributable risks:

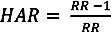
, where:
(1)

- *HAR* is the daily heat-related attributable risk
- *RR* is the calculated relative risk at each temperature from the city-specific model

Next, we calculated annual May–September heat-related mortality rates as follows:

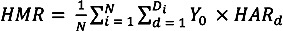
, where:
(2)

- *HMR* is the mean annual heat-related mortality rate for each time period
- *N* is the number of years in each time period (22 for the baseline and 30 for the future periods)
- *i* is an index for year in each time period
- *D_i_* is the number of days in the *i*-th year
- *d* is an index of day in the *i*-th year
- *Y_0_* is daily mortality rate (per 100,000 population) calculated using the year 2000 population and city-specific mortality rates for Boston, New York and Philadelphia from the CDC Wonder database [35]

We also calculated annual heat-related daily deaths:

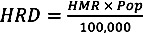
, where:
(3)

- *HRD* is the number of annual heat-related deaths in each period
- *Pop* is the population of each city using the year 2000 population data from the CDC Wonder database [35]

## 3. Results

Temperature, population and mortality summary statistics for Boston, New York City and Philadelphia are presented in Table 1. New York City is located about 190 miles from Boston and 80 miles from Philadelphia. The region spans the transition between the humid subtropical and humid continental climates. 

**Table 1:** Population, mortality and temperature statistics for Boston, New York City and Philadelphia.

City	Population (2000) ^1^	Annual/Daily Mortality Rate per 100,000 (2000) ^1^	Mean Summer^2 ^ Temperature (°C) ^3^	Mean Annual Temperature (°C) ^3^
Boston	689,807	795/2.18	19.6	10.9
New York City	8,008,278	754/2.07	21.7	13
Philadelphia	1,517,550	1167/3.2	22.4	13.4

^1^ Population and mortality rates obtained from the CDC Wonder Database [35]; ^2^ Includes data for May, June, July, August and September; ^3^ Temperature data obtained from the U.S. National Climatic Data Center [24].

**Figure 1:**
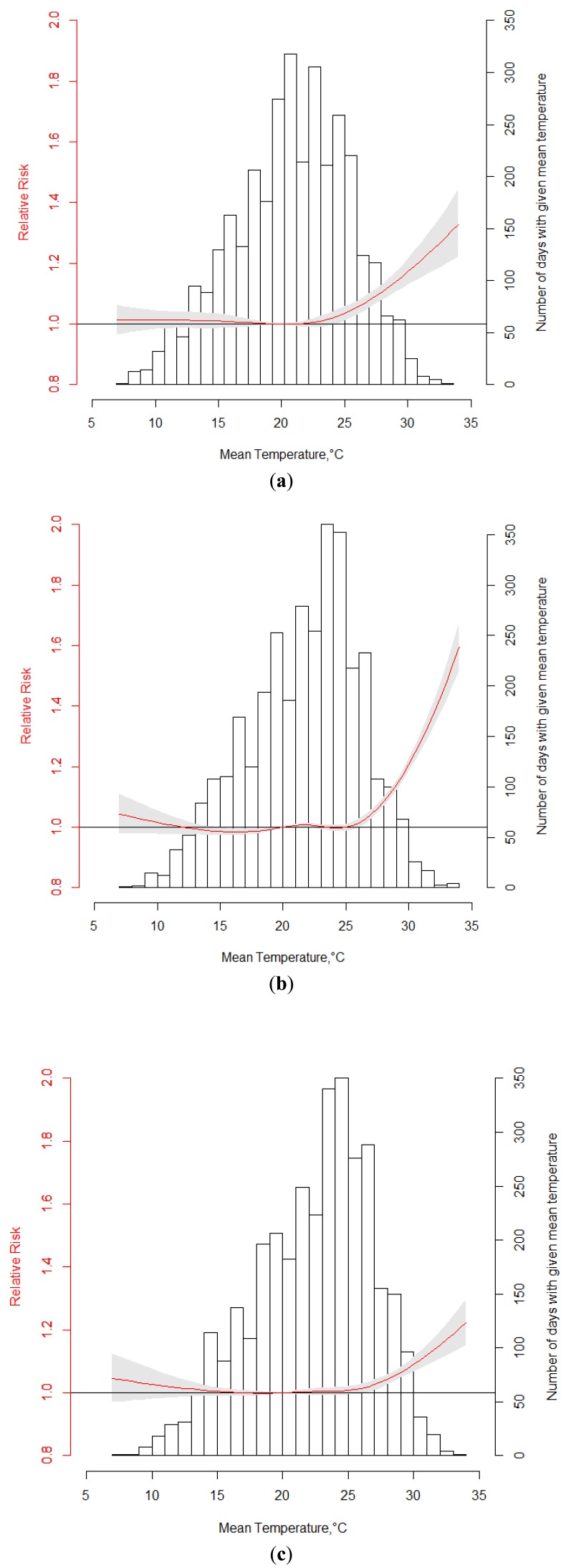
Temperature‒mortality curves of overall cumulative relative risk over four days of lag and mean summer temperature histograms for (**a**) Boston (**b**) New York City and (**c**) Philadelphia based on data between 1985 and 2006. Relative risks calculated using a distributed lag non-linear model with natural cubic splines with four degrees of freedom for the temperature and the lag and 20 °C as a reference temperature.

The summer temperature-mortality relationships derived using the non-linear distributed lag models along with summer temperature histograms are presented in Figure 1a–c. The overall structure of the temperature-mortality relationships was similar for the three cities. Also, for all cities, cumulative relative risks were slightly elevated at the lowest temperatures. There was no difference in the lag structure across the three cities (not displayed). Nonetheless, some differences were also evident. First, a heat effect was observed above around 26 °C in New York City and Philadelphia and above 24 °C in Boston. Also, mortality risk at very high temperatures was substantially more pronounced in New York City compared to Boston and Philadelphia.

**Figure 2:**
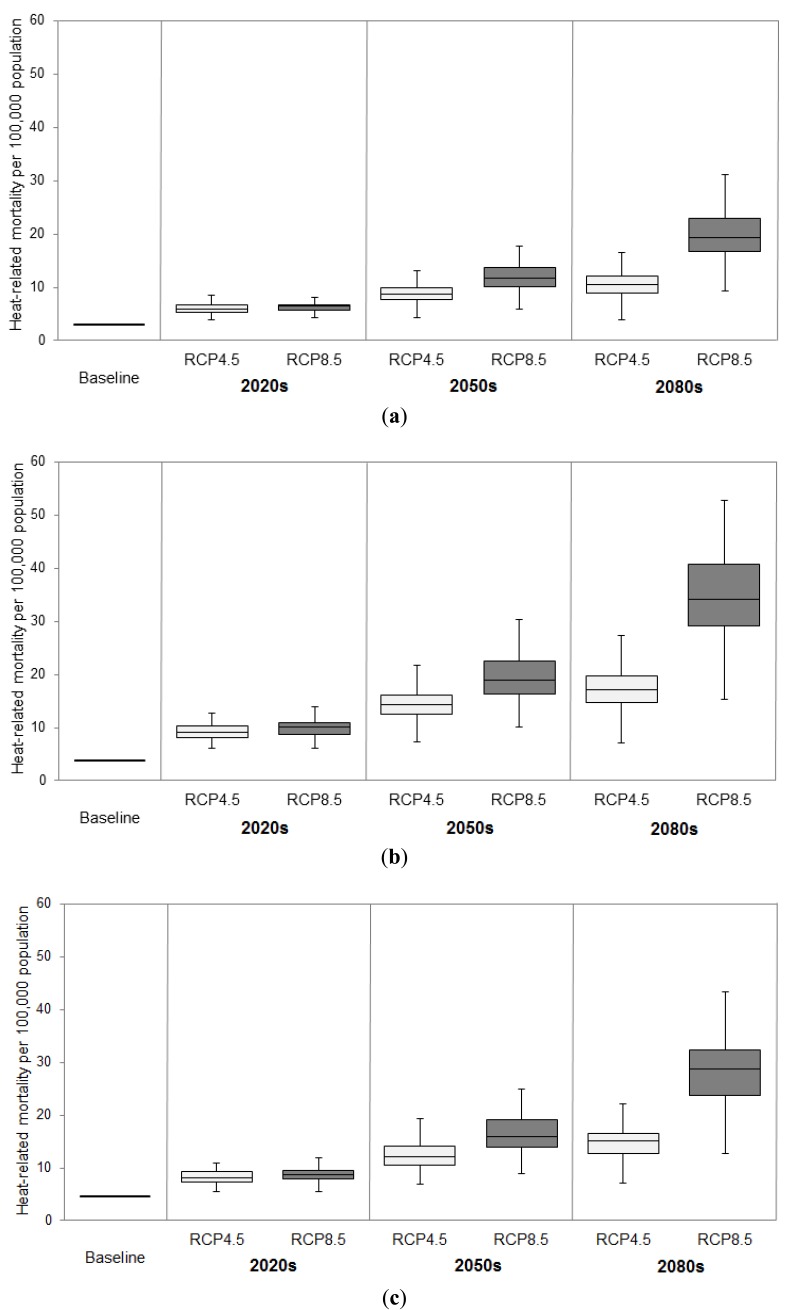
Projected annual heat-related mortality rates during the 2020s, 2050s and 2080s for (**a**) Boston (**b**) New York City and (**c**) Philadelphia, during the baseline period (1985–2006) and according to the 33 global climate models (GCMs) and two Representative Concentration Pathways (RCPs), RCP4.5 and RCP8.5. Box plots illustrate the minimum, lower quartile, median, upper quartile and maximum values across the GCMs, by period and RCP. Also displayed are the annual heat-related mortality rates computed for the baseline period between 1985 and 2006, based on observed temperatures.

Annual baseline and projected heat-related mortality rates for Boston, New York City and Philadelphia are presented in Figure 2a–c and the Table S2. Baseline heat-related mortality rates were highest in Philadelphia (4.5 per 100,000) followed by New York City (3.7 per 100,000) and Boston (2.9 per 100,000). Projected heat-related mortality rates based on the downscaled temperature projections were highest in New York City followed by Philadelphia and Boston. We first computed heat-related mortality rates for each GCM (Table S2) and then reported median values by decade and RCP. During the 2020s, median heat-related mortality rates calculated across all models and the RCP4.5 and RCP8.5, were 9.1 and 10 per 100,000, respectively, for New York City, 8 and 8.8 per 100,000 for Philadelphia and 5.9 and 6.5 per 100,000 for Boston. In the 2050s, New York City was projected to experience median mortality rates of 14.3 and 18.9 per 100,000, Philadelphia of 12.2 and 16 per 100,000 for and Boston of 8.8 and 11.7 per 100,000, for RCP4.5 and RCP8.5, respectively. By the 2080s, projected median heat-related mortality rates across all models and the RCP4.5 and RCP8.5 were 17.1 and 34.3 per 100,000 for New York City, 15.2 and 28.7 for Philadelphia, and 10.5 and 19.3 per 100,000 for Boston.

The relative increase in heat-related mortality by the end of the century was greater in New York City, followed by Boston and Philadelphia. By the 2080s under RCP4.5, the calculated heat-related mortality rates represent an over three-fold increase in Philadelphia, a nearly four-fold increase in Boston and nearly five-fold increase in New York City. By the 2080s under RCP8.5, these rates represent an over six-fold increase in heat-related mortality in Philadelphia, a nearly seven-fold increase in Boston and over nine-fold increase in New York City. 

New York City was projected to experience the greatest increase in the number of heat-related deaths due to its large population, followed by Philadelphia and Boston. Heat-related deaths are calculated using Equation (3) and the median heat-related mortality rates from Table S2. By the 2080s, the calculated mortality rates according to the RCP8.5 or RCP4.5 correspond to 2,743 or 1,336 summer heat-related deaths annually compared to 297 during the baseline period for New York City, 436 or 231 summer heat-related deaths annually compared to 68 during the baseline period for Philadelphia, and 133 or 73 summer heat-related deaths annually compared to 20 during the baseline period for Boston. 

## 4. Discussion

Characterizing the heat-mortality relationships in Boston, New York City and Philadelphia based on daily temperature and mortality data in the period between 1985 and 2006 was the first step in our assessment of future heat-related mortality in the three cities. The similarity of the heat-mortality curves across the three cities was not surprising given their close proximity and similar climates. However, the substantially higher mortality risk at very high temperatures observed in New York City during the baseline period compared to Boston and Philadelphia warrants further investigation. Based on the relative geographical location of the three cities, one might expect that the heat effect would be most pronounced in Boston where summers are the coolest followed by New York City and finally Philadelphia, where summers are the hottest overall. Several factors may be contributing to the higher historical heat-related mortality observed in New York City. First, as the biggest of the three cities, New York City may be experiencing a greater urban heat island effect, resulting in substantially higher temperatures within the city’s neighborhoods compared to New York Central Park where the temperature monitoring station is located. According to the U.S. Environmental Protection Agency, temperature in cities can be up to 12 °C higher compared to surrounding areas [36,37]; one study in New York City found that the heat island can average 4 °C and reach up to 8 °C [38]. Lack of access or underutilization of air conditioning, particularly among the New York City’s most vulnerable populations may be another important factor. The elderly and those with pre-existing medical conditions have been found to be particularly susceptible to the impacts of heat [39,40,41]. Although air conditioning prevalence has been increasing steadily in the Northeast region, a far greater percentage of homes did not have air conditioning in New York, compared to Massachusetts and Pennsylvania according to the Residential Energy Consumption Survey (RECS) carried out by the U.S. Energy Information Administration (EIA) [42]. According to the survey, as of 2009, 19.4% of New York homes did not have air conditioning compared to 12% in Massachusetts and 6.1% in Pennsylvania. Further, 6.9%, 8% and 6.1% in New York, Massachusetts and Pennsylvania, respectively, did not use existing air conditioning equipment in their homes. Data on air conditioning utilization during heat events was unfortunately not available. In a recent case review of 26 heat-related deaths in New York City with documented air conditioning data, 88% lacked air conditioning at home and the remaining 12% had air conditioning that wasn’t used for technical or other reasons [43]. To prevent heat-related mortality among individuals with a medical conditions exacerbated by heat, New York has started providing air conditioning to eligible individuals through the Home Energy Assistance Program (HEAP) [44].

After characterizing heat-related mortality in each city, we compared the city-specific baseline and projected heat-related mortality rates. An interesting finding of the analyses was the higher baseline heat-related mortality rate in Philadelphia compared to Boston and New York City. As described previously, heat-related mortality rates were derived by multiplying the temperature-specific heat-related attributable risks by city-specific mortality rates per 100,000. Thus, despite the more pronounced heat effect at very high temperature in New York City, the baseline heat-related mortality rate is higher in Philadelphia due to the city’s high mortality rate (Table 1). Nonetheless, projected heat-related mortality rates are greatest in New York City, followed by Philadelphia and Boston in each of the three future periods (Figure 2a–c). The substantial increase in heat-related mortality projected by our models in all of the three cities provides further evidence of the vulnerability to heat in the region.

The increasing number of days with moderately and very high temperatures is a main driver of the future increases heat-related mortality. For New York City, the impacts of heat are further exacerbated by the magnitude of the mortality response at very high temperatures. Finally, the choice of RCP plays a substantial role in projecting future heat-related mortality, particularly in the second half of the century. During the 2020s, estimates derived using RCP4.5 and RCP8.5 do not vary greatly. By the 2080s, however, median heat-related mortality rates calculated across all models under RCP8.5 were near twice as high as those calculated under RCP4.5. These findings illustrate the health impacts associated with the difference between scenarios in which greenhouse gas concentrations in the atmosphere/radiative forcing continue to increase (RCP8.5) or stabilize over time (RCP4.5), respectively.

Our analysis of heat-related mortality rates across the three cities illustrates the influence and interplay of the various input parameters, such as temperature-specific relative risks, mortality rates and population in each city. Thus, assumptions about each of these inputs have important impacts on the interpretation of findings. Our study has several important limitations. First, we assumed that population in each city will remain constant at the 2000 Census level. This may lead to underestimation of future impacts because urbanization will likely continue in the region throughout the century. Similarly, we assumed constant city-specific mortality rates. Mortality rates may decrease in the coming decades if life expectancy continues to increase and improvements of the overall quality of life of the population continue to take place. We also assumed that the derived temperature-mortality curves will remain unchanged throughout the century. This may not be the case since populations are likely to acclimatize to heat over time. Therefore, this approach may represent an overestimation of future impacts of heat. Nonetheless, assuming constant temperature-specific relative risks, mortality rates and population in each city allowed the estimation of potential heat-related mortality impacts due to climate change in each city. The resulting comparative assessment of projected heat-related mortality can be of value in supporting decision maker efforts to reduce heat-related vulnerability in the region.

## 5. Conclusions

We presented an assessment of the potential impacts of climate change on heat-related mortality in the three largest cities of the Northeast U.S.—Boston, New York City and Philadelphia—using the climate models and two Representative Concentration Pathways (RCPs) from the Intergovernmental Panel on Climate Change (IPCC)’s Fifth Assessment Report (AR5). To our knowledge this is the first such study.

We found that although heat-mortality curves across the three cities were similar, New York experienced a more pronounced heat effect at very high temperatures compared to Boston and Philadelphia. However, that heat-related mortality rates per 100,000 of population during the baseline period were highest in Philadelphia followed by New York City and Boston. Nonetheless, the projected heat-related mortality rates in the 2020s, 2050s and 2080s were highest in New York City, followed by Philadelphia and Boston. By the 2080s, these rates represent an over three-fold increase in Philadelphia, a nearly four-fold increase in Boston and nearly five-fold increase in New York City under RCP4.5 and an over six-fold increase in Philadelphia, a nearly seven-fold increase in Boston and over nine-fold increase in New York under RCP8.5. The presented estimates allow a comparative assessment of the potential impacts of climate change on heat-related mortality in the three cities that can be of value to various stakeholders interested in developing strategies to reduce these impacts and building climate change resilience in the urban Northeast region.

## Supplementary Files

Supplementary File 1 Supplementary Information (PDF, 113 KB)
