# Supplementary material for: Projected Heat-Related Mortality in the U.S. Urban Northeast
Source: Int J Environ Res Public Health. 2013 Dec 3;10(12):6734–47. doi: 10.3390/ijerph10126734 (PMC3881138; doi:10.3390/ijerph10126734)
Supplement: Supplementary File 1 — Supplementary Information (PDF, 113 KB) [file ijerph-10-06734-s001.pdf]

Projected Heat-Related Mortality in the U.S. Urban Northeast

Table S1. IPCC AR5 GCMs used in this study. The models were developed by 22 modeling centers (left column). Some centers support multiple GCMs, and/or versions of their GCM.

Modeling Center	Institute ID	Model Name	Atmospheric Resolution (lat × lon)
Commonwealth Scientific and Industrial Research Organization (CSIRO) and Bureau of Meteorology (BOM), Australia	CSIRO-BOM	ACCESS1.0<br>ACCESS1.3	1.25 × 1.875<br>1.25 × 1.875
Beijing Climate Center, China Meteorological Administration	BCC	BCC-CSM1.1<br>BCC-CSM1.1(m)	2.8 × 2.8<br>1.1 × 1.1
College of Global Change and Earth System Science, Beijing Normal University	GCESS	BNU-ESM	2.8 × 2.8
Canadian Centre for Climate Modelling and Analysis	CCCMA	CanESM2	2.8 × 2.8
National Center for Atmospheric Research	NCAR	CCSM4	0.9 × 1.25
Community Earth System Model Contributors	NSF-DOE-NCAR	CESM1(BGC)<br>CESM1(CAM5)	0.9 × 1.25<br>0.9 × 1.25
Centro Euro-Mediterraneo per l Cambiamenti Climatici	CMCC	CMCC-CM	0.75 × 0.75
Centre National de Recherches Météorologiques/Centre Européen de Recherche et Formation Avancée en Calcul Scientifique	CNRM-CEFRACS	CNRM-CM5	1.4 × 1.4
Commonwealth Scientific and Industrial Research Organization in collaboration with Queensland Climate Change Centre of E × cellence	CSIRO-QCCE	CSIRO-Mk3.6.0	1.9 × 1.9
LASG, Institute of Atmospheric Physic, Chinese Academy of Sciences and CESS, Tsinghua University	LASG-CESS	FGOALS-g2	2.8 × 2.8
The First Institute of Oceanography, SOA, China	FIO	FIO-ESM	2.8 × 2.8
NOAA Geophysical Fluid Dynamics Laboratory	NOAA GFDL	GFDL-CM3<br>GFDL-ESM2G<br>GFDL-ESM2M	2.0 × 2.5<br>2.0 × 2.5<br>2.0 × 2.5
NASA Goddard Institute for Space Studies	NASA GISS	GISS-E2-R	2.0 × 2.5

Table S1. *Cont.*

Modeling Center	Institute ID	Model Name	Atmospheric Resolution (lat × lon)
National Institute of Meteorological Research/Korea Meteorological Administration	NIMR/KMA	HadGEM2-AO	1.25 × 1.875
Met Office Hadley Centre (additional HadGEM2-ES realizations contributed by Instituto Nacional de Pesquisas Espaciais)	MOHC (additional realizations by INPE)	HadGEM2-CC<br>HadGEM2-ES	1.25 × 1.875<br>1.25 × 1.875
Institute for Numerical Mathematics	INM	INM-CM4	1.5 × 2.0
Institut Pierre-Simon Laplace	IPSL	IPSL-CM5A-LR<br>IPSL-CM5A-MR<br>IPSL-CM5B-LR	1.9 × 3.75<br>1.3 × 2.5<br>1.9 × 3.75
Japan Agency for Marine-Earth Science and Technology, Atmosphere and Ocean Research Institute (The University of Tokyo), and National Institute for Environmental Studies)	MIROC	MIROC-ESM<br>MIROC-ESM-CHEM	2.8 × 2.8<br>2.8 × 2.8
Atmosphere and Ocean Research Institute (The University of Tokyo), National Institute for Environmental Studies, and Japan Agency for Marine-Earth Science and Technology	MIROC	MIROC5	1.4 × 1.4
Max Planck Institute for Meteorology	MPI-M	MPI-ESM-MR<br>MPI-ESM-LR	1.9 × 1.9<br>1.9 × 1.9
Meteorological Research Institute	MRI	MRI-CGCM3	1.1 × 1.1
Norwegian Climate Centre	NCC	NorESM1-M<br>NorESM1-ME	1.9 × 2.5<br>1.9 × 2.5

Table S2. Heat-related mortality rates per 100,000 population during the baseline period between 1985 and 2006 for Boston, New York City and Philadelphia (**a**) and projected annual heat-related mortality rates per 100,000 population during the 2020s, 2050s and 2080s for Boston, New York City and Philadelphia according to the each of the 33 global climate models (GCMs) and the two Representative Concentration Pathways (RCPs) used in this study: (**b**) $RCP_{4.5}$ and (**c**) $RCP_{8.5}$.

(**a**)

Baseline (1985–2006)		
**Boston**	**NYC**	**Philadelphia**
2.9	3.7	4.5

(**b**)

GCM	RCP 4.5								
	**Boston**			**NYC**			**Philadelphia**		
	**2020s**	**2050s**	**2080s**	**2020s**	**2050s**	**2080s**	**2020s**	**2050s**	**2080s**
access1-0	7.7	9.9	11.7	11.7	15.9	18.8	9.3	13.8	16.3
access1-3	6.2	8.5	10.7	8.8	12.5	15.4	7.9	11.4	13.7
bcc-csm1-1	5.2	7.8	9.3	8.0	14.8	14.7	7.4	13.4	13.7
bcc-csm1-1-m	5.8	7.7	8.9	9.2	14.1	15.4	9.6	13.6	15.9
bnu-esm	7.8	11.0	14.2	12.5	17.7	22.8	10.0	14.7	18.3
canesm2	6.8	10.9	13.0	10.5	16.3	20.0	10.5	14.7	17.5
ccsm4	5.5	8.1	8.2	8.3	13.1	14.6	7.6	10.7	11.6
cesm1-bgc	5.4	8.2	8.3	9.1	15.1	15.1	7.5	11.7	11.7
cesm1-cam5	6.6	9.5	12.2	10.3	16.1	20.0	8.9	12.3	16.5
cmcc-cm	6.6	8.8	11.3	8.8	14.4	19.1	8.0	12.6	15.8
cnrm-cm5	4.9	7.4	9.7	7.3	11.1	15.4	6.8	9.0	12.7
csiro-mk3-6-0	5.4	8.9	10.5	8.3	14.1	17.1	7.7	11.9	15.3
fgoals-g2	6.6	9.9	10.8	10.4	18.0	19.4	9.3	14.1	15.2
fio-esm	4.0	4.4	3.8	6.1	7.3	7.0	5.6	6.9	7.1
gfdl-cm3	7.3	12.9	16.6	11.4	24.4	29.7	10.1	19.6	24.6
gfdl-esm2g	5.0	7.0	5.8	6.4	10.5	9.0	6.3	9.4	8.8
gfdl-esm2m	4.7	5.9	6.8	7.8	9.7	10.8	6.8	8.0	8.9
giss-e2-r	5.2	6.4	6.5	8.1	10.1	11.4	7.3	9.5	10.3
hadgem2-ao	9.2	14.1	15.1	12.7	20.0	24.4	10.9	16.7	20.9
hadgem2-cc	6.8	10.9	14.6	9.6	16.9	24.3	9.1	14.3	20.0
hadgem2-es	6.5	11.0	15.4	10.8	18.6	24.8	9.9	15.5	20.4
inmcm4	4.2	4.8	6.1	6.1	8.0	10.1	5.7	6.9	8.8
ipsl-cm5a-lr	6.4	9.0	11.6	9.1	15.3	18.8	7.9	12.8	15.7
ipsl-cm5a-mr	6.4	8.9	10.3	9.3	15.9	17.5	8.5	13.2	15.6
ipsl-cm5b-lr	5.5	8.3	10.1	8.1	13.4	15.9	7.2	11.1	12.9
mirco-esm	6.6	11.7	13.4	10.3	18.6	22.2	8.9	15.3	17.5
miroc-esm-chem	7.0	11.3	12.6	10.3	16.2	19.8	9.6	14.5	16.6

Table S2. (b) *Cont.*

GCM	RCP 4.5								
	Boston			NYC			Philadelphia		
	2020s	2050s	2080s	2020s	2050s	2080s	2020s	2050s	2080s
miroc5	5.9	9.0	9.8	9.3	14.3	16.2	8.3	12.2	13.8
mpi-esm-lr	5.9	8.0	10.2	9.0	12.5	14.0	7.8	10.5	12.7
mpi-esm-mr	5.7	7.3	9.6	8.1	11.6	15.7	8.1	10.6	13.8
mri-cgcm3	4.7	7.0	6.9	6.9	10.7	10.8	6.3	9.0	10.0
noresm1-m	6.0	8.9	11.1	9.3	13.1	17.8	8.3	11.9	15.2
noresm1-me	5.6	8.7	11.8	8.6	14.2	19.3	7.9	12.0	15.1
**median**	**5.9**	**8.8**	**10.5**	**9.1**	**14.3**	**17.1**	**8.0**	**12.2**	**15.2**

(c)

GCM	RCP 8.5								
	Boston			NYC			Philadelphia		
	2020s	2050s	2080s	2020s	2050s	2080s	2020s	2050s	2080s
access1-0	6.5	12.5	20.9	10.3	19.2	37.7	9.5	17.3	32.0
access1-3	5.9	9.7	16.7	8.3	15.4	27.0	8.1	13.9	23.8
bcc-csm1-1	6.6	10.8	18.2	9.9	17.3	33.2	9.5	15.6	28.7
bcc-csm1-1-m	6.6	12.3	17.0	11.5	22.0	32.6	10.6	19.2	30.7
bnu-esm	7.3	14.2	24.5	11.5	22.8	42.6	10.1	19.4	32.3
canesm2	7.4	15.8	26.8	11.6	23.3	41.4	11.2	21.4	36.7
ccsm4	5.5	10.1	16.0	9.3	15.9	30.7	8.1	14.0	23.8
cesm1-bgc	6.3	10.1	15.9	10.0	16.4	29.1	8.2	14.4	23.4
cesm1-cam5	6.8	12.2	19.9	10.5	20.0	37.7	9.1	16.6	29.2
cmcc-cm	6.5	10.1	18.2	10.0	17.3	35.3	8.8	15.4	30.7
cnrm-cm5	5.8	10.0	16.9	8.8	16.9	27.4	7.7	14.0	22.4
csiro-mk3-6-0	5.4	11.3	18.8	8.8	19.1	33.7	7.8	16.5	26.9
fgoals-g2	7.1	13.6	21.0	11.2	24.9	40.6	10.0	20.0	30.5
fio-esm	4.3	5.9	10.8	6.8	11.1	20.6	5.9	9.9	17.8
gfdl-cm3	7.3	17.8	30.4	13.9	30.3	52.8	12.5	24.9	43.5
gfdl-esm2g	5.6	8.6	15.4	8.7	14.3	24.6	7.7	12.2	21.6
gfdl-esm2m	5.6	8.9	13.0	9.5	15.3	25.0	7.7	12.2	19.7
giss-e2-r	5.6	7.8	11.8	8.5	13.4	19.9	7.3	11.8	17.2
hadgem2-ao	6.8	15.6	28.2	9.0	22.6	42.5	9.0	17.8	35.0
hadgem2-cc	7.3	15.7	30.6	11.0	25.2	49.0	9.3	21.5	40.1
hadgem2-es	5.9	15.8	31.2	9.7	23.1	51.4	9.6	21.1	40.5
inmcm4	4.2	6.3	9.2	6.1	10.1	15.2	5.5	8.9	12.7
ipsl-cm5a-lr	6.4	12.3	22.9	9.9	20.1	40.8	8.8	17.4	32.3
ipsl-cm5a-mr	6.5	12.2	22.0	10.2	19.9	40.8	8.8	17.7	34.6
ipsl-cm5b-lr	6.9	11.5	19.9	10.3	18.2	34.7	8.9	15.1	27.2
mirco-esm	8.2	14.2	25.8	13.0	23.5	43.7	10.7	19.9	33.3
miroc-esm-chem	7.6	15.8	27.7	10.9	23.5	45.8	9.5	19.5	35.4
miroc5	6.2	12.8	19.3	10.3	18.9	32.5	8.3	15.5	25.9

Table S2. (c) *Cont.*

GCM	RCP 8.5								
	Boston			NYC			Philadelphia		
	2020s	2050s	2080s	2020s	2050s	2080s	2020s	2050s	2080s
mpi-esm-lr	6.8	12.7	22.0	11.4	21.1	38.2	9.2	17.1	29.8
mpi-esm-mr	5.9	11.3	19.9	8.3	18.4	34.3	8.0	16.0	28.6
mri-cgcm3	4.9	8.5	12.6	7.9	13.3	20.3	6.4	10.9	17.3
noresm1-m	6.0	10.8	19.0	9.3	17.7	32.5	7.9	15.0	25.5
noresm1-me	6.7	11.7	18.7	10.4	18.2	32.7	8.5	14.7	26.1
**median**	**6.5**	**11.7**	**19.3**	**10.0**	**18.9**	**34.3**	**8.8**	**16.0**	**28.7**

© 2013 by the authors; licensee MDPI, Basel, Switzerland. This article is an open access article distributed under the terms and conditions of the Creative Commons Attribution license (http://creativecommons.org/licenses/by/3.0/).
